# Associations Between Infant Screen Use, Electroencephalography Markers, and Cognitive Outcomes

**DOI:** 10.1001/jamapediatrics.2022.5674

**Published:** 2023-01-30

**Authors:** Evelyn C. Law, Meredith X. Han, Zhuoyuan Lai, Shuping Lim, Zi Yan Ong, Valerie Ng, Laurel J. Gabard-Durnam, Carol L. Wilkinson, April R. Levin, Anne Rifkin-Graboi, L. Mary Daniel, Peter D. Gluckman, Yap Seng Chong, Michael J. Meaney, Charles A. Nelson

**Affiliations:** 1 Department of Paediatrics, Yong Loo Lin School of Medicine, National University of Singapore, Singapore; 2 Khoo Teck Puat–National University Children’s Medical Institute, National University Health System, Singapore; 3 Singapore Institute for Clinical Sciences, Agency for Science, Technology and Research, Singapore; 4 Center for Cognitive and Brain Health, Northeastern University, Boston, Massachusetts; 5 Division of Developmental Medicine, Boston Children’s Hospital, Boston, Massachusetts; 6 Division of Neurology, Boston Children’s Hospital, Boston, Massachusetts; 7 Centre for Research in Child Development, National Institute of Education, Singapore; 8 Department of Child Development, KK Women’s and Children’s Hospital, Singapore; 9 Academic Medicine Department, Duke-NUS Medical School, Singapore; 10 Liggins Institute, University of Auckland, Grafton, Auckland, New Zealand; 11 Department of Obstetrics and Gynaecology, National University Health System and Yong Loo Lin School of Medicine, National University of Singapore, Singapore; 12 Ludmer Centre for Neuroinformatics and Mental Health, Department of Psychiatry, McGill University, Montreal, Quebec, Canada; 13 McGill University and Douglas Mental Health University Research Centre, Montreal, Quebec, Canada; 14 Department of Psychiatry, Harvard Medical School, Boston, Massachusetts

## Abstract

**Importance:**

Research evidence is mounting for the association between infant screen use and negative cognitive outcomes related to attention and executive functions. The nature, timing, and persistence of screen time exposure on neural functions are currently unknown. Electroencephalography (EEG) permits elucidation of the neural correlates associated with cognitive impairments.

**Objective:**

To examine the associations between infant screen time, EEG markers, and school-age cognitive outcomes using mediation analysis with structural equation modeling.

**Design, Setting, and Participants:**

This prospective maternal-child dyad cohort study included participants from the population-based study Growing Up in Singapore Toward Healthy Outcomes (GUSTO). Pregnant mothers were enrolled in their first trimester from June 2009 through December 2010. A subset of children who completed neurodevelopmental visits at ages 12 months and 9 years had EEG performed at age 18 months. Data were reported from 3 time points at ages 12 months, 18 months, and 9 years. Mediation analyses were used to investigate how neural correlates were involved in the paths from infant screen time to the latent construct of attention and executive functioning. Data for this study were collected from November 2010 to March 2020 and were analyzed between October 2021 and May 2022.

**Exposures:**

Parent-reported screen time at age 12 months.

**Main Outcomes and Measures:**

Power spectral density from EEG was collected at age 18 months. Child attention and executive functions were measured with teacher-reported questionnaires and objective laboratory-based tasks at age 9 years.

**Results:**

In this sample of 437 children, the mean (SD) age at follow-up was 8.84 (0.07) years, and 227 children (51.9%) were male. The mean (SD) amount of daily screen time at age 12 months was 2.01 (1.86) hours. Screen time at age 12 months contributed to multiple 9-year attention and executive functioning measures (η^2^, 0.03-0.16; Cohen *d*, 0.35-0.87). A subset of 157 children had EEG performed at age 18 months; EEG relative theta power and theta/beta ratio at the frontocentral and parietal regions showed a graded correlation with 12-month screen use (*r* = 0.35-0.37). In the structural equation model accounting for household income, frontocentral and parietal theta/beta ratios partially mediated the association between infant screen time and executive functioning at school age (exposure-mediator β, 0.41; 95% CI, 0.22 to 0.59; mediator-outcome β, −0.38; 95% CI, −0.64 to −0.11), forming an indirect path that accounted for 39.4% of the association.

**Conclusions and Relevance:**

In this study, infant screen use was associated with altered cortical EEG activity before age 2 years; the identified EEG markers mediated the association between infant screen time and executive functions. Further efforts are urgently needed to distinguish the direct association of infant screen use compared with family factors that predispose early screen use on executive function impairments.

## Key Points

**Question:**

To what extent is the association between infant screen use and cognitive impairments mediated by electroencephalography markers?

**Findings:**

In this birth cohort study involving 437 children, the use of a mediation analysis embedded in a structural education model provided evidence that electrocortical activity in the frontocentral and parietal brain regions mediated the association between infant screen use and later executive function impairments.

**Meaning:**

Screen use during infancy may contribute to variations in neural activities implicated in the development of high-order cognitive skills.

## Teaser

This cohort study analyzes data for children from the population-based study Growing Up in Singapore Toward Healthy Outcomes (GUSTO) to examine the associations between infant screen time, electroencephalography markers, and school-age cognitive outcomes using a mediation analysis.

## Introduction

Since the advent of mobile electronic devices, infants aged between 6 and 18 months are exposed to 2 to 3 hours of screen time per day.^1,2,3,4^ This amount far exceeds the policy statement and recommendation by the American Academy of Pediatrics, which discourages screen media use before age 18 months except for video chatting.^5^ Studies suggest an association between screen use and negative cognitive outcomes. Of particular importance are findings of associations between screen use in early childhood (ages 6 months to 4 years) and impairments of attention and executive functions.^6,7,8,9,10,11^

Executive functions represent a collection of higher-order cognitive skills essential for self-regulation, learning, and academic achievement, as well as mental health.^12,13^ These functions develop rapidly over the first years of life in concert with the prefrontal cortex and are highly susceptible to environmental influences.^14,15,16^ Infants exposed to screens are particularly vulnerable to executive function deficits due to their difficulty processing information on 2-dimensional screens, also called video deficit.^17^ The need to comprehend challenging screen content, particularly content designed for older children and adults that is unfamiliar and fantastical in nature, requires tremendous cognitive resources and processing.^6,7,8,18^ This kind of processing relies heavily on attention primarily through the sensory pathways of the brain (ie, bottom-up attention), which leaves inadequate allocation of resources for prefrontal, top-down attention and typical development of executive functions.^19^

Emerging neuroimaging studies in preschool-age children have demonstrated associations between exposure to screen-based media and alterations in white matter tracts important for executive functioning.^20,21^ To date, little is known about potential neural correlates in infants who are most vulnerable to later executive function deficits due to the aforementioned video deficit. Furthermore, it is unclear whether these deficits persist into school age. The nature and timing of screen time on the underlying neural processes mediating cognitive changes are also unknown.

Electroencephalography (EEG) is a powerful and widely available tool that has been used extensively to identify neural correlates of various cognitive functions. Resting EEG data over the midfrontal and parietal regions have been used to delineate potential neural mechanisms of attention and executive function.^22,23^ Converging data reveal that an increase in low-frequency powers (eg, slow theta wave) and a higher relative ratio of theta to beta powers (also called theta/beta ratio) are both neural correlates of poorer attentional control.^24,25^ A previous study found a correlation between screen exposure and higher functional connectivity in EEG theta waves; however, EEG was collected in only 14 children ages 4 to 6 years after a 9-hour screen-based storytelling intervention.^26^ We aimed to establish the association between screen use at age 12 months and known neural signals of poorer attentional control at age 18 months, particularly in the theta frequency band and in the theta/beta ratio, and to then examine whether these differences in early electrocortical activity contribute to the variances in attention and executive function outcomes at age 9 years.

## Methods

### Design and Oversight

Growing Up in Singapore Toward Healthy Outcomes (GUSTO) is a population-based, prospective cohort with the aim of understanding perinatal and early influences on long-term health outcomes. Women 18 years and older across all socioeconomic backgrounds were recruited at their first trimester of pregnancy from 2 main public hospitals in Singapore between June 2009 and December 2010.^27^ Data were collected from November 2010 to March 2020. Race and ethnicity were reported by both parents. In Singapore, the 3 major ethnicities included Chinese, Indian, and Malay. Mother-child dyads were followed up throughout pregnancy and beyond. The study was approved by the SingHealth Centralised Institutional Review Board and the National Health Group Domain-Specific Review Board. Mothers provided written informed consent prior to enrollment in the cohort, and all children assented to the study at 7 years of age. We followed the Strengthening the Reporting of Observational Studies in Epidemiology (STROBE) reporting guideline.

### Participants

This cohort consisted of 506 mother-child dyads who were invited to complete the study measures when children were ages 12 months and 9 years. Children who were born preterm (<37 weeks), born small for gestational age (<10th percentile on the World Health Organization growth charts), part of a twin pregnancy, or those with major neurological conditions were excluded (n = 28). At age 9 years, 437 children (86.3%) had complete behavioral data for analyses. Reasons for the missing data (n = 41) are listed in eFigure 1 in Supplement 1. A subset of 157 children were part of the EEG sample, as previously reported.^28^

### Exposure and Measures

Monthly household income was stratified into 4 groups: less than SGD 2000 (US $1478), SGD 2000 to 3999 (US $1478-$2955), SGD 4000 to 5999 (US $2956-$4433), and SGD 6000 or greater (≥US $4434). The group with monthly income less than SGD 2000 represented those who likely received governmental financial subsidy based on absolute criterion in 2010.^29^ In Singapore, economic deprivation and psychological ill effects are often reported in families receiving subsidies, with estimates of 14% to 31% endorsing severe to extremely severe scores on the Depression, Anxiety and Stress Scale (DASS-21).^30^ When their child was aged 12 months, parents were asked to report the amount of time on average that the child spent on screens per day on weekdays, Saturdays, and Sundays in the past 1 month. We used the total hours of screen time watched over the entire week and divided it by 7 days to give the amount of screen time per day. Parents were asked the same question on their child’s screen time at 5 time points between ages 12 months and 54 months. Screen time data across time points were moderately to strongly correlated (*r* = 0.40-0.51) in our cohort.^31^

At age 18 months, a subset of the cohort underwent EEG, which was acquired in a dimly lit, electrically shielded room with 128-channel Geodesic Sensor Nets connected to a DC-coupled amplifier (Net Amp 300, Electrical Geodesic). Resting EEG was recorded continuously for 3 minutes while the child was seated on the lap of a caregiver and looking at bubbles being blown in the room. Data were processed using the Harvard Automated Processing Pipeline for EEG (HAPPE) embedded within the Batch EEG Automated Processing Platform.^32,33^ The processing involved 1-Hz high-pass and 100-Hz low-pass filtering, removal of 50-Hz line noise, bad channel detection, wavelet-enhanced independent component analysis (ICA), ICA with artifact removal through Multiple Artifact Rejection Algorithm, interpolation of bad channels, and re-referencing to the average reference.^34,35^ Data were segmented into contiguous 2-second segments, and segment rejection was carried out via HAPPE criteria.^35^ The eTable 1 in Supplement 1 shows our data quality metrics. Based on our metrics, EEG data from 7 participants were removed.

A fast Fourier transform with the multitaper method was used to calculate the power spectrum on each 2-second segment. Power was parsed into frequency bands (delta, 2-3.99 Hz; theta, 4-5.99 Hz; low alpha, 6-8.99 Hz; high alpha, 9-12.99 Hz; beta, 13-29.99 Hz; gamma, 30-44.99 Hz). The summed absolute power across these frequencies represented the total power. The mean summed absolute power in each frequency band across all 2-second segments was calculated and normalized by a log-10 transformation. Relative power for each band was calculated as a percentage of the total power. The theta/beta ratio was calculated by dividing the power within theta frequencies by the power within beta frequencies. Given our attention and executive function outcomes, the regions of interest were the frontocentral and parietal regions, which included 16 and 4 electrodes, respectively (eFigure 2 in Supplement 1).

### Outcomes

Objective executive function assessments were administered using the Developmental Neuropsychological Assessment, second edition (NEPSY-II), which incorporated the 3 core executive function components, naming inhibition, shifting, and working memory.^12,36^ The inhibition task in the NEPSY-II is a Stroop task requiring predominantly inhibition in one condition and shifting in another condition. The word interference task requires working memory, the ability to remember and manipulate information in mind. Higher scores on the inhibition scaled score, shifting scaled score, and working memory recall scaled score indicated better competency in executive functioning. The *t* scores from the Attention Problems scale in the Child Behavior Checklist (CBCL) and the General Executive Control Problems scale from the Behavior Rating Inventory of Executive Function, second edition (BRIEF-2), were obtained from teachers.^37,38^ Higher scores in CBCL and BRIEF-2 reflected more problems in attention and executive functioning.

### Statistical Analyses

Analyses using the χ^2^ test and 1-way analysis of variance were conducted to compare categorical and linear variables across groups and to examine differences due to loss to follow-up between invited families, study sample, and EEG subsample. Linear regression models confirmed whether screen time at age 12 months was associated with attention and executive functions at age 9 years. Covariates in the model, including household income, birth weight, smoking exposure during pregnancy, child sex, and negative maternal mental health during pregnancy, were all associated with executive functioning in prior literature.^29,39,40^ We examined whether neural changes in the frequency bands responded in a dose-response manner based on the amount of screen time at age 12 months. We then tested for correlations between screen time duration and 18-month absolute and relative EEG power measures at the frontocentral and parietal regions. Mediation analyses using maximum likelihood estimation within a structural equation modeling framework were used to investigate the extent to which neural correlates were involved in the paths from infant screen time to the latent construct of attention and executive functioning. Data were analyzed between October 2021 and May 2022 using Stata version 15.1 (StataCorp) and Mplus version 12 (Muthén & Muthén). Topography maps were visualized using MNE Python.^41^

## Results

Table 1 shows the demographic characteristics of GUSTO children across 3 groups. The mean (SD) age at follow-up was 8.84 (0.07) years, and 227 children (51.9%) were male. No significant differences in demographic characteristics were found between the groups.

**Table 1.** Demographic Characteristics of the Study Samples

	No./total No. (%)		
	Invited families (n = 506)	Study sample (n = 437)	EEG subsample (n = 150)
Sex			
Male	269/506 (53.2)	227/437 (51.9)	81/150 (54.0)
Female	237/506 (46.8)	210/437 (48.1)	69/150 (46.0)
Smoke exposure during pregnancy	77/500 (15.4)	84/435 (19.3)	27/146 (18.5)
Monthly household income, SGD (US$)			
<2000 (1478)	116/493 (23.5)	95/424 (22.4)	31/138 (22.5)
2000-3999 (1478-2955)	119/493 (24.1)	106/424 (25.0)	34/138 (24.6)
4000-5999 (2956-4433)	124/493 (25.2)	109/424 (25.7)	35/138 (26.1)
≥6000 (4434)	134/493 (27.2)	114/424 (26.9)	37/138 (26.8)
Maternal education level			
High school and below	149/500 (29.8)	138/431 (32.0)	47/146 (32.2)
Diplomas/certificates	177/500 (35.4)	166/431 (38.5)	57/146 (39.0)
University and above	174/500 (34.8)	127/431 (29.5)	42/146 (28.8)
Ethnicity			
Chinese	285/506 (56.3)	250/437 (57.2)	76/150 (50.7)
Malay	144/506 (28.5)	122/437 (27.9)	51/150 (34.0)
Indian	77/506 (15.2)	65/437 (14.9)	23/150 (15.3)
Maternal age at delivery, mean (SD), y	31.25 (5.11)	31.42 (5.11)	31.26 (5.28)
Screen time, mean (SD), h/d	2.01 (1.85)	2.12 (1.94)	2.34 (1.87)
Birth weight, mean (SD), kg	3.12 (0.43)	3.17 (0.39)	3.15 (0.40)
Gestational age, median (IQR), wk	38.86 (38.00-39.71)	38.93 (38.00-39.71)	38.78 (38.14-39.50)

Abbreviations: EEG, electroencephalography; SGD, Singapore dollars.

### Screen Time, Attention, and Executive Functioning

The mean (SD) amount of screen time was 2.01 (1.86) hours per day at 12 months. After adjustment for covariates, screen time at 12 months was independently associated with 9-year teacher-reported and objectively measured attention and executive functioning (Table 2). Household income was a significant covariate in the task-based outcomes (eTable 2 in Supplement 1). In the regression models, every hour increase in screen time was associated with a 0.30 to 0.56 decrease in scaled score of each task, which amounted to a reduction of 1.42 scaled score points (SD, 0.47) when all 3 components of executive function were accounted for.

**Table 2.** Infant Screen Time and Its Association With 9-Year Attention and Executive Functioning in Regression Models^a^

Cognitive domain	Cognitive problem or skill	Report or task	*t*	Coefficient (95% CI)	SE	*η^2^*
Attention	Attention problems^b^	Teacher report	2.87	1.06 (0.31 to 1.81)	0.37	0.09
Executive functioning	General executive control problems^c^	Teacher report	2.30	1.72 (0.21 to 3.23)	0.75	0.08
	Inhibition^d^	Task	−2.96	−0.56 (−0.94 to −0.19)	0.23	0.16
	Shifting^d^	Task	−3.12	−0.56 (−0.91 to −0.20)	0.18	0.16
	Working memory recall^d^	Task	−2.12	−0.30 (−0.58 to −0.02)	0.14	0.03

Abbreviations: BRIEF-2, Behavior Rating Inventory of Executive Function, second edition; CBCL, Child Behavior Checklist; NEPSY-II, Developmental Neuropsychological Assessment, second edition.

^a^ Adjusted for covariates including household income, birth weight, smoking exposure during pregnancy, child sex, and antenatal maternal mental health factor.

^b^ *t* Scores (mean [SD], 50 [10]) from the CBCL Attention Problems scale.

^c^ *t* Score from the BRIEF-2 General Executive Control Problems scale.

^d^ NEPSY-II scaled scores (mean [SD], 10 [3]).

### Screen Time and Neural Correlates

**Figure 1.**
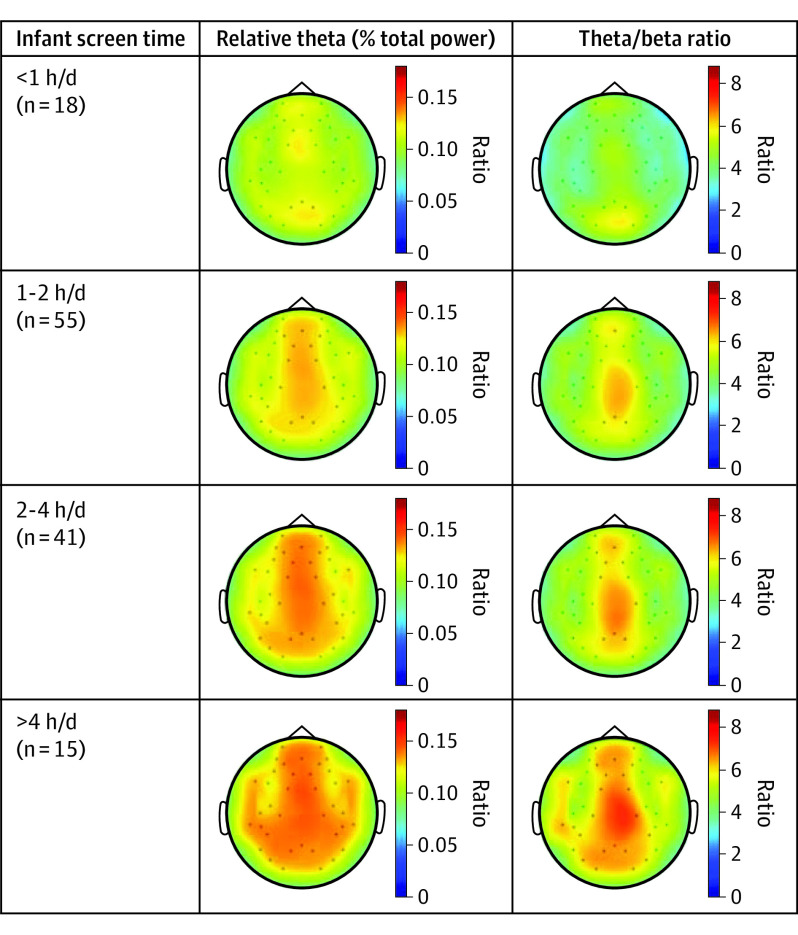
Brain Topographic Maps of Postulated Neural Correlates Based on Infant Screen Time per Day

We examined the associations between screen time, relative theta, theta/beta ratio, and outcomes in a correlation matrix (Table 3). All the neural correlates implicated in attention, including relative theta and theta/beta ratio, were correlated with screen time at 12 months in a linear fashion. To visualize this relationship, topographic maps of the average brain activation were produced and stratified into 4 groups of daily screen time duration for ease of interpretation: less than 1 hour (n = 18), 1 to 2 hours (n = 55), 2 to 4 hours (n = 41), and more than 4 hours (n = 15) (Figure 1). As screen time increased, the relative theta power was higher, which was mirrored in the theta/beta ratio. Using the same scale, topographic maps were compared with 4 groups of household income. Results produced a similar, yet less prominent, gradient compared with infant screen time (eFigure 3 in Supplement 1).

**Table 3.** Bivariate Correlations Between Screen Time, Relative Theta, Theta/Beta Ratio, and Laboratory-Based Executive Function Outcomes

	β (95% CI)							
	1	2	3	4	5	6	7	8
1. 12-mo Screen time	1 [Reference]							
2. Frontocentral relative theta	0.36 (0.14 to 0.57)	1 [Reference]						
3. Parietal relative theta	0.37 (0.15 to 0.59)	0.90 (0.86 to 0.95)	1 [Reference]					
4. Frontocentral theta/beta	0.36 (0.16 to 0.57)	0.86 (0.81 to 0.91)	0.79 (0.69 to 0.89)	1 [Reference]				
5. Parietal theta/beta	0.35 (0.14 to 0.57)	0.80 (0.75 to 0.85)	0.88 (0.85 to 0.91)	0.90 (0.85 to 0.95)	1 [Reference]			
6. Task: inhibition	−0.29 (−0.42 to −0.14)	−0.17 (−0.38 to 0.05)	−0.19 (−0.38 to 0.01)	−0.24 (−0.42 to −0.05)	−0.23 (−0.42 to −0.06)	1 [Reference]		
7. Task: shifting	−0.29 (−0.42 to −0.15)	−0.18 (−0.38 to 0.02)	−0.18 (−0.38 to 0.02)	−0.24 (−0.40 to −0.03)	−0.21 (−0.39 to −0.03)	0.86 (0.83 to 0.88)	1 [Reference]	
8. Task: working memory	−0.15 (−0.28 to −0.03)	−0.10 (−0.31 to 0.10)	−0.18 (−0.37 to 0.02)	−0.24 (−0.41 to −0.04)	−0.25 (−0.45 to −0.05)	0.28 (0.19 to 0.36)	0.32 (0.23 to 0.40)	1 [Reference]

### EEG Power and Outcomes

Relative theta and theta/beta ratios of the frontocentral and parietal electrodes were associated with each of the laboratory-based executive function tasks (Table 3). The higher the relative theta and theta/beta ratios, the poorer the performance in executive functioning. None of the EEG power parameters were associated with attention and executive functioning reported by teachers (eTable 3 in Supplement 1).

### Structural Equation Model With Mediation Analysis

**Figure 2.**
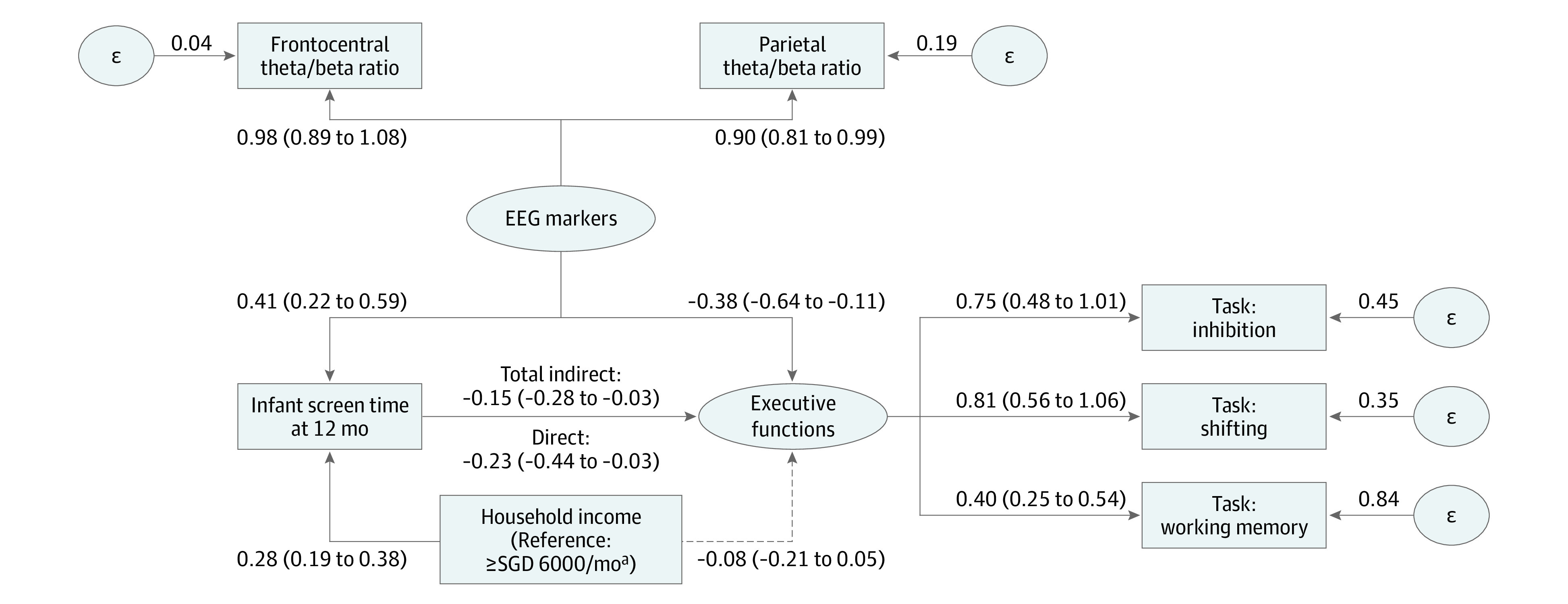
Frontocentral and Parietal Theta/Beta Ratios Partially Mediated the Path From Screen Time to Executive Functions Numbers are β (95% CI). Executive function tasks were assessed using the Developmental Neuropsychological Assessment, second edition (NEPSY-II). The ε represents measurement errors of the endogenous variables in the structural equation models. EEG indicates electroencephalography; SGD, Singapore dollars. ^a^US $4434.

As confirmed in GUSTO and in the regression models, socioeconomic status was consistently associated with infant screen time.^2^ Thus, we added household income as an exogenous variable in the structural equation model. We found a direct path from infant screen time to the latent outcome construct consisting of all 3 laboratory-based executive function tasks (exposure-outcome β, −0.23; 95% CI, −0.44 to −0.03). Each of the 4 targeted mediators was tested individually, of which frontocentral and parietal theta/beta ratios provided indirect paths (eFigure 4A and B in Supplement 1). The final model included both theta/beta ratios as a latent mediator (Figure 2). Together, the EEG markers provided an indirect path (β, −0.15; 95% CI, −0.28 to −0.03) between the exposure to the criterion outcome, which accounted for 39.5% of the total effects (β, −0.38; 95% CI, −0.56 to −0.21). The fit indices were exceptional (χ^2^ = 5.86; Akaike information criterion, 8492.18; comparative fit index >0.99; root mean square error of approximation <0.001; standardized root mean square residual = 0.019). In summary, frontocentral and parietal theta/beta ratios partially mediated the path from screen time at age 12 months to the latent executive function outcome at age 9 years.

## Discussion

Our study provides evidence for the persisting longitudinal association between infant screen time at age 12 months and attention and executive functioning outcomes at 9 years of age. The outcome measures were teacher reports and objective laboratory tasks. Both corroborate real-world manifestations of observable impairments, although teacher reports may be limited by subjectivity or cultural interpretation of behaviors. In short, increased screen time in infancy is associated with impairments in cognitive processes critical for health, academic achievement, and future work success. However, the findings from this cohort study do not prove causation. Screen time likely represents a measurable contextual characteristic of a family or a proxy for the quality of parent-child interaction. Replication of this study’s findings and randomized clinical trials are warranted.

We also document a positive “dose-response” association between infant screen time and cortical EEG correlates of attention and executive functioning. This association is detectable in a stepwise manner from 1 hour to more than 4 hours of screen time per day. The mediation analyses demonstrate cortical EEG activity, namely the theta/beta ratio, as a plausible frontoparietal-mediated pathway from infant screen time to poor executive function. The mapping of the EEG signals to the frontoparietal regions is of critical importance, as these regions are core neural substrates involved in working memory, orienting attention (ie, a shift in attention toward the salient stimulus), and executive control subfunctions of attention.^42,43^

Our findings provide evidence for the potential value of EEG during early childhood in understanding executive function impairments later in childhood. Attention and executive functions are difficult to reliably assess in early childhood and may not be apparent until the academic demands increase during formal school years.^44^ EEG activities associated with later outcomes offer a means of determining risks and facilitating earlier interventions. Because executive functions develop rapidly in conjunction with the prefrontal cortex and are highly trainable skills, timing interventions during this period of neuroplasticity and before neuronal circuits stabilize must be considered.^15,45^ While the clinical utility of these electrophysiological measures in the diagnosis of attention-deficit/ hyperactivity disorder is debated,^25,46^ this study is interested in using these measures to show a brain-behavior relationship that functions across a continuum in the context of infant screen time.^47^

Our analyses underscore the association between infant screen time, cortical activity, and cognitive function. It is important to point out that screen use may be a proxy for cognitive impoverishment due to the displacement of social interactions in real life, which are “experience-expectant” inputs needed to facilitate executive function development.^15,48^ The sensitive caregiving and reciprocal interactions between caregivers and infants remain crucial in regulating the physiology of the infant and in the building of cognitive, social, and affective competencies.^16^ On the other hand, there is reason to assume a direct effect of screen time on neurodevelopment. Very young media consumers have developmentally appropriate but incongruous response to stimuli presented on 2-dimensional screens, coupled with reduced ability to attend selectively to relevant stimuli via this medium.^17^ To interpret repeated and multiple novel streams of sensory inputs through the device, infants expend large amounts of mental effort, particularly through activating the orienting reflexes. They do so without the capacity to modulate such efforts, which impedes adequate development of the executive control system during the early years.^14,49^

### Limitations

This study has limitations that warrant consideration. Screen time at 12 months of age was reported by parents and not an objective measure. At that point, precise recording of screen use via moment-to-moment capture and machine learning, now referred to as screenome, was still in development. Time spent on each type of electronic device was also not collected. In 2010, handheld devices were beginning to surface in Singapore, and 97% of families were using television alone as the main source of screen time.^2^ While it was assumed that television viewing decreased with the advent of handheld devices, television viewing continued to account for a large proportion of screen time in children younger than 5 years.^4^ Although the amount of screen time of GUSTO infants was found to be comparable with other countries in this study, it was unclear whether infant screen time changed during the recent COVID-19 pandemic lockdowns. Older children in our Singapore cohorts were shown to use screens more often, particularly in online chats.^50^ Hence, generalizability may be limited because of these new trends in screen use.

In addition, our study was not designed to inform on the issue of screen content. However, some argued whether the cognitive immaturity of infants rendered content less relevant compared with older children. This study was limited to identifying individual cognitive risks and lacked the inclusion of contextual influences, including parent-child interactions and language stimulation activities. We recognized the importance of ecological context of early media use and prioritized these topics in ongoing studies.^51^ Lastly, families in the cohort reported household income in categories, which prevented us from examining this socioeconomic indicator with a greater level of granularity. Nevertheless, the arbitrary cutoffs of household income into 4 categories remained a strong correlate of infant screen time, which echoed prior literature on the need to address this issue with at-risk families.

## Conclusions

We used a longitudinal cohort to define the enduring associations between screen time in infancy and cognitive skills in late childhood. Infant screen use was associated with altered cortical EEG activity before age 2 years, a time when real-life problems related to attention could not be reliably confirmed. Furthermore, the identified EEG markers mediated the association between infant screen time and executive functions. Given the pervasiveness of infant screen use, our findings have public health implications on a population level. Further efforts are urgently needed to distinguish the direct association of infant screen use vs family factors that predispose early screen use on executive function impairments.

## Notes

Supplement 1. **eTable 1. **The Harvard Automated Processing Pipeline for EEG (HAPPE) processing quality metrics **eTable 2. **Monthly household income and its association with 9-year executive function tasks **eTable 3. **Bivariate correlations between screen time, relative theta, theta/beta ratio, and teacher-reported outcomes **eFigure 1. **Flowchart of participant inclusion and exclusion **eFigure 2.** Processed frontocentral (grey) and parietal (black) EEG electrodes **eFigure 3. **Brain topographic maps of 18-month relative theta and theta/beta ratio based on monthly household income **eFigure 4. **Frontocentral and parietal theta/beta ratio individually mediated the path from screen time to executive functions

Supplement 2. Data sharing statement
