# Supplementary material for: Associations Between Infant Screen Use, Electroencephalography Markers, and Cognitive Outcomes
Source: JAMA Pediatr. 2023 Jan 30;177(3):311–8. doi: 10.1001/jamapediatrics.2022.5674 (PMC9887532; doi:10.1001/jamapediatrics.2022.5674)
Supplement: Supplement 1. — eTable 1. The Harvard Automated Processing Pipeline for EEG (HAPPE) processing quality metrics eTable 2. Monthly household income and its association with 9-year executive function tasks eTable 3. Bivariate correlations between screen time, relative theta, theta/beta ratio, and teacher-reported outcomes eFigure 1. Flowchart of participant inclusion and exclusion eFigure 2. Processed frontocentral (grey) and parietal (black) EEG electrodes eFigure 3. Brain topographic maps of 18-month relative theta and theta/beta ratio based on monthly household income eFigure 4. Frontocentral and parietal theta/beta ratio individually mediated the path from screen time to executive functions [file jamapediatr-e225674-s001.pdf]

Supplemental Online Content

Law EC, Han MX, Lai Z, et al. Associations between infant screen use, electroencephalography markers, and cognitive outcomes. *JAMA Pediatr*. Published online January 30, 2023. doi:10.1001/jamapediatrics.2022.5674

eTable 1. The Harvard Automated Processing Pipeline for EEG (HAPPE) processing quality metrics

eTable 2. Monthly household income and its association with 9-year executive function tasks

eTable 3. Bivariate correlations between screen time, relative theta, theta/beta ratio, and teacher-reported outcomes

eFigure 1. Flowchart of participant inclusion and exclusion

eFigure 2. Processed frontocentral (grey) and parietal (black) EEG electrodes

eFigure 3. Brain topographic maps of 18-month relative theta and theta/beta ratio based on monthly household income

eFigure 4. A) Frontocentral and **B)** Parietal theta/beta ratio individually mediated the path from screen time to executive functions

This supplemental material has been provided by the authors to give readers additional information about their work.

© 2023 Law EC et al. *JAMA Pediatr.*

eTable 1. The Harvard Automated Processing Pipeline for EEG (HAPPE) processing quality metrics

**Quality metrics**	**Mean**	***SD***
Length of raw EEG (seconds)	188.70	28.80
Good channels (%)	91.17	5.28
Rejected independent components per recording period (%)	35.99	12.68
EEG variance retained (%)	70.07	16.42
Mean retained artifact probability	0.11	0.04
Median retained artifact probability	0.06	0.05

© 2023 Law EC et al. *JAMA Pediatr.*

eTable 2. Monthly household income and its association with 9-year executive function tasks

**Executive Function Domain**	**Household income (Ref: <SGD 2000/month)**								
	**SGD 2000-3999 Coefficient (95% CI)**	***SE***	***p***	**SGD 4000-5999 Coefficient (95% CI)**	***SE***	***p***	**≥SGD 6000 Coefficient (95% CI)**	***SE***	***p***
Inhibition Scaled Score	-0.33 (-1.39, 0.74)	0.54	0.55	0.50 (-0.62, 1.62)	0.57	0.38	**2.89 (0.50, 5.29)**	**1.21**	**0.018**
Shifting Scaled Score	0.44 (-0.76, 1.64)	0.61	0.47	**2.23 (0.97, 3.50)**	**0.64**	**0.001**	**3.38 (2.13, 4.63)**	**0.63**	**<0.001**
Working Memory Scaled Score	0.40 (-0.32, 1.11)	0.36	0.28	0.70 (-0.05, 1.45)	0.38	0.07	**1.60 (0.87, 2.34)**	**0.37**	**<0.001**

© 2023 Law EC et al. *JAMA Pediatr.*

eTable 3. Bivariate correlations between screen time, relative theta, theta/beta ratio, and teacher-reported outcomes

	1	2	3	4	5	6	7
1. 12-month screen time	1						
2. Frontocentral relative theta	**0.36** (0.14, 0.57)	1					
3. Parietal relative theta	**0.37** (0.15, 0.59)	**0.90** (0.86, 0.95)	1				
4. Frontocentral theta/beta	**0.36** (0.16, 0.57)	**0.86** (0.81, 0.91)	**0.79** (0.69, 0.89)	1			
5. Parietal theta/beta	**0.35** (0.14, 0.57)	**0.80** (0.75, 0.85)	**0.88** (0.85, 0.91)	**0.90** (0.85, 0.95)	1		
6. Teacher-rated attention problems	**0.30** (0.07, 0.54)	0.06 (-0.29, 0.42)	0.03 (-0.30, 0.36)	0.14 (-0.29, 0.56)	0.09 (-0.29, 0.47)	1	
7. Teacher-rated executive control problems	**0.33** (0.10, 0.57)	-0.15 (-0.52, 0.21)	0.02 (-0.34, 0.39)	-0.09 (-0.56, 0.39)	0.02 (-0.44, 0.48)	**0.61** (0.48, 0.74)	1

© 2023 Law EC et al. *JAMA Pediatr.*

eFigure 1. Flowchart of participant inclusion and exclusion

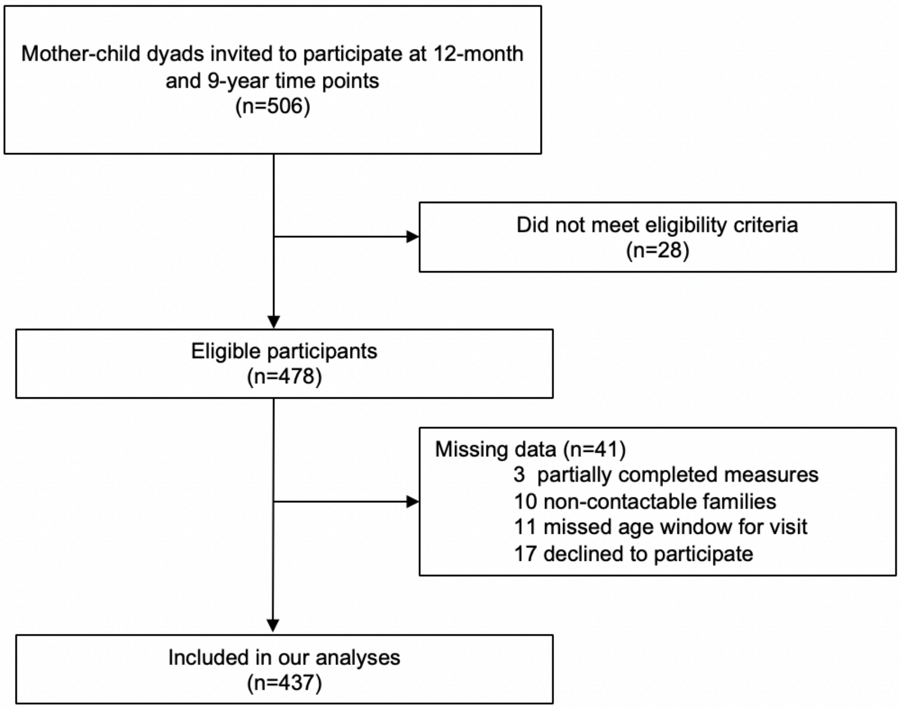

© 2023 Law EC et al. *JAMA Pediatr.*

eFigure 2. Processed frontocentral (grey) and parietal (black) EEG electrodes

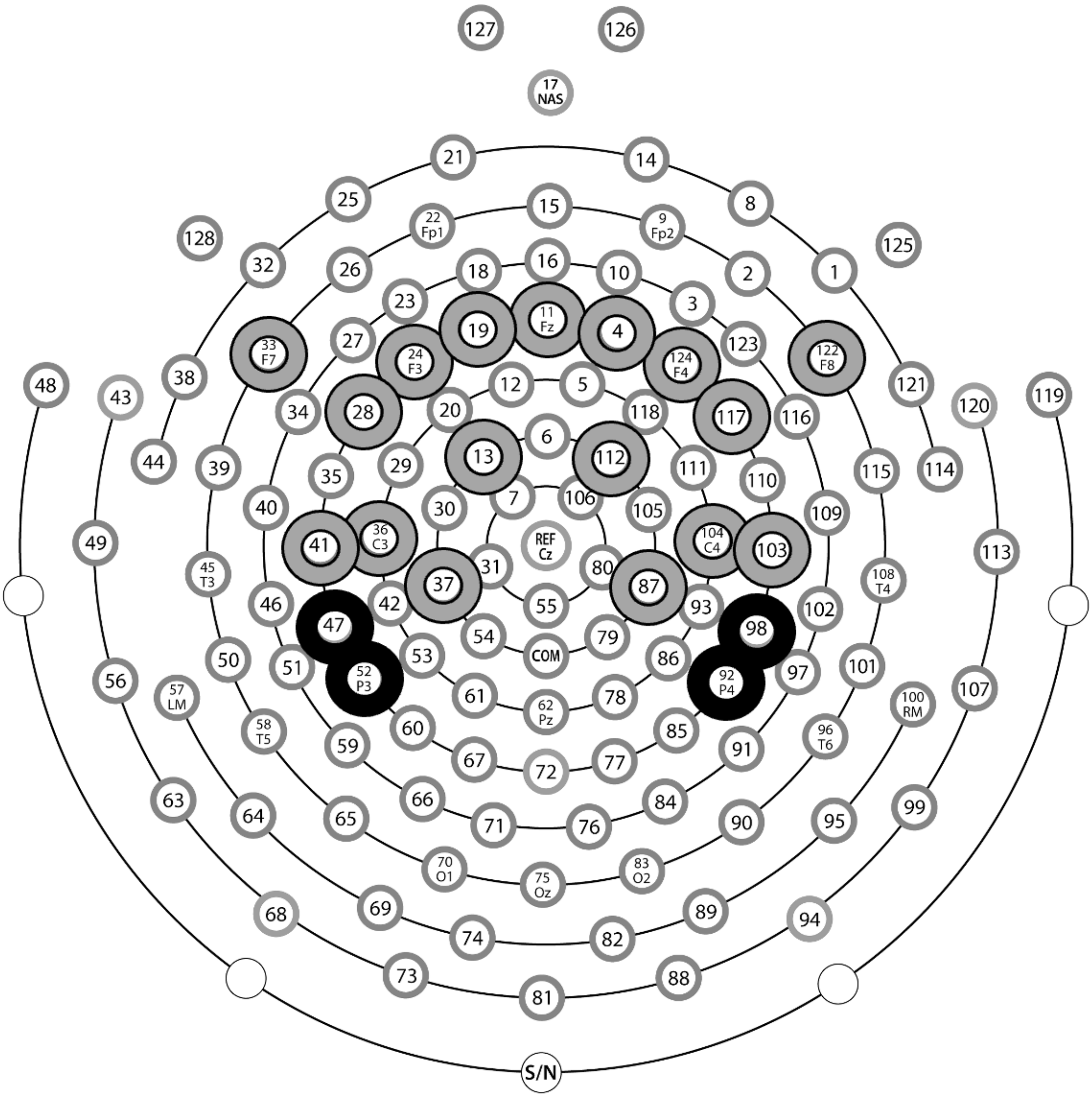

© 2023 Law EC et al. *JAMA Pediatr.*

eFigure 3. Brain topographic maps of 18-month relative theta and theta/beta ratio based on monthly household income

**Household Income**	**Relative Theta (% Total Power)**	**Theta/Beta Ratio**
<SGD 2000/month (n=27)	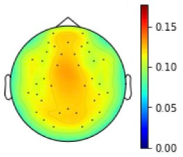	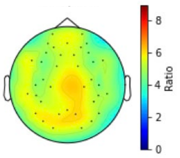
SGD 2000-3999/month (n=54)	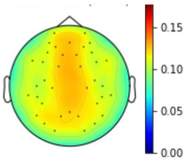	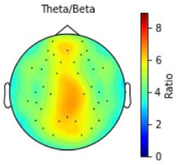
SGD 3000-5999/month (n=31)	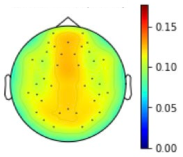	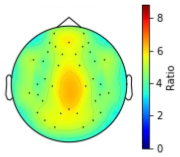
≥SGD 6000/month (n=37)	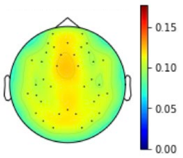	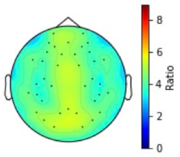

© 2023 Law EC et al. *JAMA Pediatr.*

eFigure 4. A) Frontocentral and **B)** Parietal theta/beta ratio individually mediated the path from screen time to executive functions

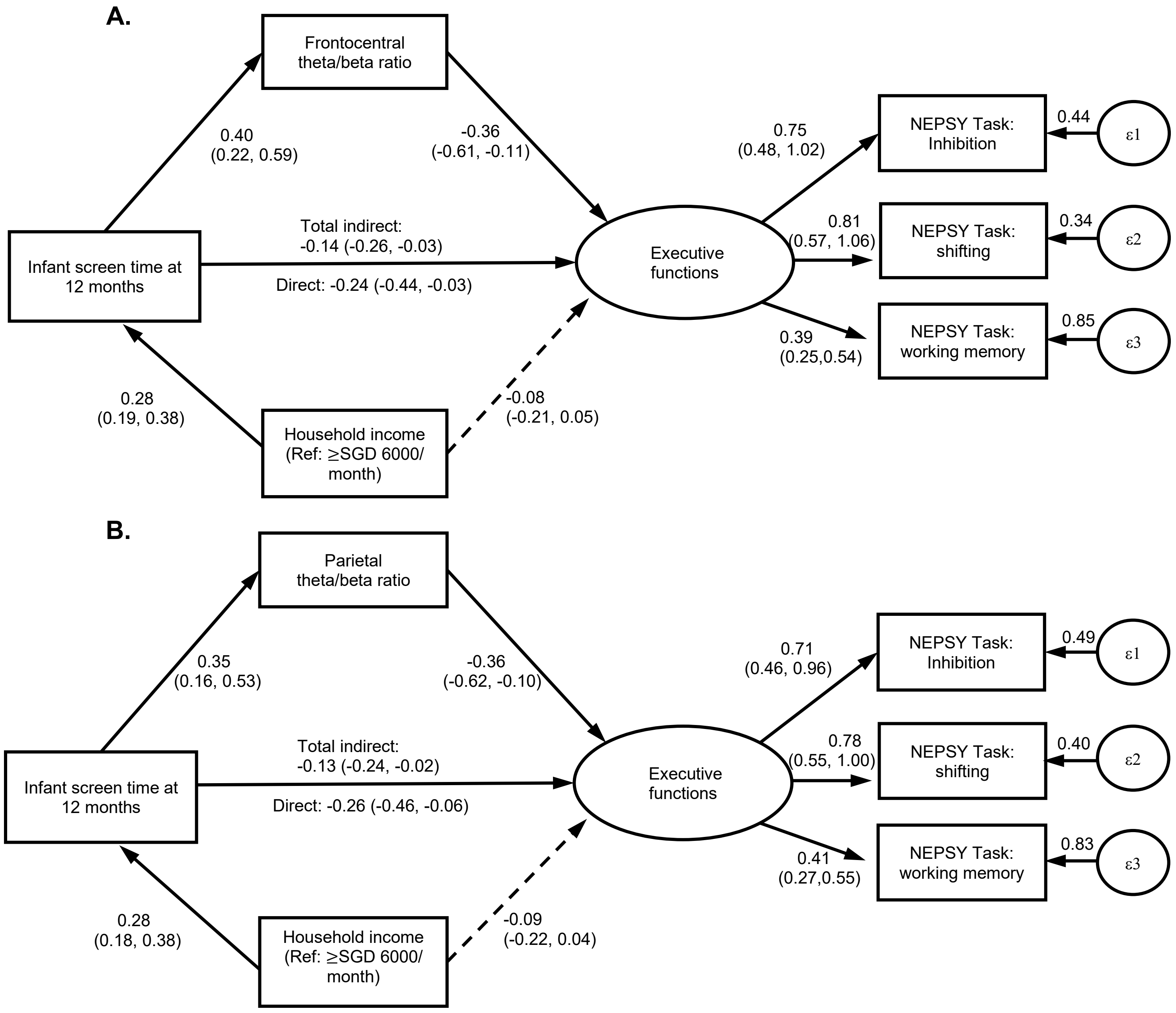

© 2023 Law EC et al. *JAMA Pediatr.*
