# Supplementary material for: Associations Between Infant Screen Use, Electroencephalography Markers, and Cognitive Outcomes
Source: JAMA Pediatr. 2023 Jan 30;177(3):311–8. doi: 10.1001/jamapediatrics.2022.5674 (PMC9887532; doi:10.1001/jamapediatrics.2022.5674)
Supplement: Supplement 2. — Data sharing statement [file jamapediatr-e225674-s002.pdf]

Data Sharing Statement

Law. Associations Between Infant Screen Use, Electroencephalography Markers, and Cognitive Outcomes. *JAMA Pediatr*. Published January 30, 2023.
doi:10.1001/jamapediatrics.2022.5674

Data
Data available: No

Additional Information
Explanation for why data not available: This cohort study requires ethics approval for each specific research question before data may be shared. The data used in this cohort are described in https://gustodatavault.sg/. The data will be made available to researchers who provide a methodologically sound proposal.
